# Homogeneous and highly dispersed Ni–Ru on a silica support as an effective CO methanation catalyst

**DOI:** 10.1039/c7ra13147j

**Published:** 2018-01-09

**Authors:** Yi Liu, Wei Sheng, Zhanggui Hou, Yi Zhang

**Affiliations:** State Key Laboratory of Organic–Inorganic Composites, Department of Chemical Engineering, Beijing University of Chemical Technology Beijing 100029 China yizhang@mail.buct.edu.cn +86 10 64436991; CNOOC Research Institute of Refining and Petrochemicals Beijing 102209 China houzhg2@cnooc.com.cn +86 10 84528796

## Abstract

The highly dispersed SiO_2_-supported nickel-based catalysts for CO methanation were prepared by an ethylene glycol (EG) modified wet-impregnation method. The results indicate that the highly dispersed 20Ni/SiO_2_ (EG) catalyst realized good stability and higher catalytic activity than the catalyst obtained from a non-pretreated silica support (20Ni/SiO_2_) in CO methanation, due to the smaller nickel particles and strong nickel–silica interaction. By the addition of a small amount of noble metal promoter (Ru, Pt, Pd), the catalytic activity for CO methanation was further improved dramatically and follows the order Ru > Pt > Pd. The added noble metal promoter enhanced the reduction of the nickel oxide by spill-over-hydrogen during reduction treatment, and provided more active species for the methanation reaction, resulting in 7 times higher CO conversion than the non-pretreated 20Ni/SiO_2_ catalyst. The 20Ni–0.5Ru/SiO_2_ (EG) catalyst presents superb catalytic performance in CO methanation with high activity (CO conv. 80.2%) as well as high methane selectivity (90.3%) at 275 °C without any deactivation during 50 h reaction. The obtained catalysts were characterized by XRD, TG/DTA, TEM, XPS, TPR, H_2_ chemisorption, and *in situ* DRIFTS.

## Abstract 2

The highly dispersed SiO_2_-supported nickel-based catalysts for CO methanation were prepared by an ethylene glycol (EG) modified wet-impregnation method.

## Graphical abstract

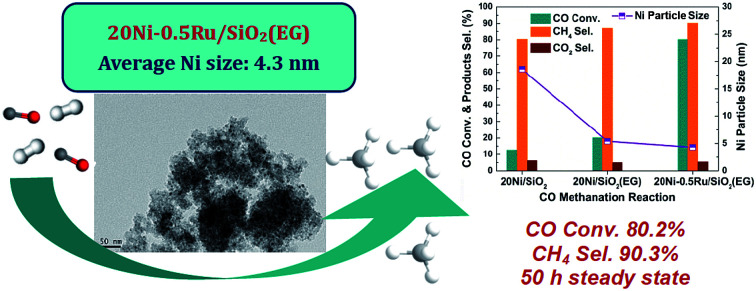

## Introduction

Strict emission legislation for the combustion of fuels requires the development of clean technologies, minimizing the environmental impact.^1^ A particularly interesting energy carrier is natural gas due to its low carbon to hydrogen ratio, advantageous combustion properties, the existing pipeline grid for transportation and the well-established technologies. In recent years, the debate of the finiteness of fossil resources and high CO_2_ emissions associated with their combustion caused increasing attention to be focussed on the research related to synthetic natural gas (SNG) production from renewable biomass,^2^ coke oven gas (COG)^3^ or syngas from coal or wood.^4^ CO methanation is an important reaction for the production of SNG.

Although supported nickel-based catalysts have been recognized as the most appropriate catalysts for CO methanation, it also exhibits some problems, such as carbon deposition or sintering of the active components Ni, which results in low activity and eventual catalyst deactivation.^5,6^ Many reports suggested that the activity and stability of the supported Ni catalysts are strongly influenced by the amount of Ni metal loading,^7,8^ the size of the dispersed Ni metal particles,^9–12^ metal–support interactions,^13,14^ and the composition of the support.^15^ It is believed that the Ni dispersion and its chemical state on the support may play a key role in its catalytic performance,^5,16^ which means the control of surface species in catalyst preparation is needed for further understanding.

Recently, we developed a simple and general method for preparing a highly dispersed supported metal catalyst by modification of a silica surface with ethylene glycol (EG) before the impregnation of metal precursors.^17–19^ This modified surface of the silica support significantly adjusted the surface functional groups of the silica support, and resulted in high dispersion of supported metal or metal oxide.^19^

Hence, in the present work, the highly dispersed silica supported Ni-based catalysts were prepared by surface modification of silica support. The smaller Ni particle size and strong Ni–support interaction was expected to reduced carbon deposition and nickel sintering, contributing to high activity and excellent stability of Ni/SiO_2_ catalyst in CO methanation. Furthermore, small amount of noble metal (Ru, Pt, Pd) promoter was added in Ni catalyst in order to improve the activity and stability in methanation reaction. All obtained nickel catalysts were characterized by XRD, TG/DTA, TEM, H_2_-TPR, H_2_ chemisorption, XPS and *in situ* DRIFT.

## Experimental

### Catalyst preparation

#### Preparation of unpromoted Ni-based catalysts

The catalysts were prepared by aqueous incipient wetness impregnation method. The loading of nickel were 20 wt% for all the catalysts. Commercially available silica gel (pore volume 1.061 mL g^−1^, pore diameter 6.7 nm, BET = 451 m^2^ g^−1^) was used as support in this study. Before the impregnation of Ni precursor, the silica gel (5 g) was treated with ethylene glycol (5.305 mL) for 1 h at 50 °C by equivalent-volume incipient-wetness impregnation method. Then the samples were dried in air at 120 °C for 12 h. The aqueous solution (5.305 mL) of nickel nitrate (Ni(NO_3_)_2_·6H_2_O, 6.2 g) was impregnated onto pretreated silica support (5 g) by incipient-wetness impregnation method followed by drying in air at 120 °C for 12 h. After that, the samples were calcinated at 400 °C for 2 h. The catalyst was marked as 20Ni/SiO_2_ (EG). For comparison, the catalysts obtained from non-pretreated silica support were marked as 20Ni/SiO_2_.

#### Preparation of Ru, Pt and Pd promoted Ni-based catalysts

The Ru-promoted Ni-based catalyst was prepared as described above by incipient wetness impregnations of aqueous solutions of nickel nitrate and ruthenium(iii) chloride (Sigma-Aldrich Co.) on silica gel supports. The loading of nickel was 20 wt% and the molar ratios of Ru/Ni were 0.025 for catalyst samples. The preparation procedure was as described for the unpromoted catalysts synthesized with the impregnation technique. The catalyst was marked as 20Ni–0.5Ru/SiO_2_ (EG). Similarly, the Pt-promoted and Pd-promoted catalysts were synthesized with the same procedure, and the palladium(ii) nitrate (Pd(NO_3_)_2_, Sigma-Aldrich Co.) and diamminedinitritoplatinum(ii) solution (Pt(NH_3_)_2_(NO_2_)_2_, Sigma-Aldrich Co.) were used as noble metal precursor. The catalyst was marked as 20Ni–0.5Pd/SiO_2_ (EG) and 20Ni–0.5Pt/SiO_2_ (EG).

For comparison, the noble metal promoted catalysts obtained from non-pretreated silica support were also prepared with the same procedure and marked as 20Ni–0.5Ru/SiO_2_, 20Ni–0.5Pd/SiO_2_, and 20Ni–0.5Pt/SiO_2_.

### Catalyst characterization

X-ray diffraction (XRD) patterns of the passivated and used catalysts were recorded on D/max2500VB2+/PC X-ray diffractometer using graphite monochromatized Cu Kα radiation (*λ* = 0.15406 nm). The morphologies and sizes of the passivated and used samples were observed by transmission electron microscope (TEM, JEOL 2100F). The samples were ultrasonically dispersed in ethanol and deposited on a carbon-enhanced copper grid. X-ray photoelectron spectrum (XPS) of the calcined catalyst was performed on VG Scientific ESCALAB 250 spectrometer to investigate the chemical state of surface cobalt species. The spectra were excited by the monochromatized Al Kα source (1486.6 eV).

H_2_-temperature programmed reduction (H_2_-TPR) experiments were carried out in a quartz tube reactor using 0.05 g calcined catalysts. The reducing gas, a mixture of 10% H_2_ diluted by Ar, was fed *via* a mass flow controller at 30 mL min^−1^ and the temperature was increased from 100 °C to 800 °C at a rate of 8 °C min^−1^. The effluent of reactor passed through a 5 Å molecular sieve trap to remove produced water, before reaching TCD.

H_2_ chemisorption experiments for passivated catalysts were performed in a static mode at 100 °C using a conventional volumetric apparatus (FINESORB-3010, FINETEC). Research grade gases (H_2_: 99.9995%) were used without further purification. Typically, 0.1 g of catalyst was used. Before adsorption of H_2_, the catalysts, which were previously reduced by H_2_ and passivated, were treated in H_2_ at 400 °C for 1 h, followed by evacuation. H_2_ adsorption isotherms were measured at 100 °C.

The thermal analysis (TG/DTA) was carried out under a dry air flow of 50 mL min^−1^ using DTG-60 system (Shimadzu) from 25 °C to 800 °C with a rate of 5 °C min^−1^.

*In situ* diffuse reflectance infrared Fourier transform (DRIFT) spectra were recorded with a Vertex 70V spectrometer (Bruker) equipped with a liquid nitrogen-cooled MCT detector (resolution 2 cm^−1^), using an *in situ* cell with CaF_2_ windows. About 15 mg of the sample, which was previously reduced by H_2_ and passivated, was loaded into the cell. Prior to CO adsorption, the sample was *in situ* treated with a H_2_ (30 mL min^−1^) gas flow at 400 °C for 1 h, followed by purging with a N_2_ (30 mL min^−1^) gas flow at the same temperature for 0.5 h, and then was cooled to 30 °C. CO (30 mL min^−1^) was introduced into the cell at room temperature for 0.5 h. After the catalysts were flushed by a N_2_ (30 mL min^−1^) gas flow for 0.5 h to remove the physical adsorption CO, the spectra of adsorbed CO were collected.

For characterization of passivated catalysts, the catalysts were pre-reduced at 400 °C for 10 h using pure H_2_ (99.999%) and then passivated by 1% O_2_ in N_2_ at room temperature to form a metal oxide layer on the surface of metal particle, in order to preventing oxidation of nickel metal when the catalysts were exposed to air.

### Catalyst evaluation tests

Catalytic activity evaluation was carried out in a continuous-flow 7 mm I.D. fixed-bed quartz tubular reactor at ambient pressure. The quartz tubular reactor was heated in a muffle furnace. The reaction temperature of catalyst bed could be monitored by a K-type thermocouple placed in the middle of the furnace. About 0.1 g catalysts diluted with 0.2 g quartz sand (20–40 mesh) was packed in reactor. Prior to reaction, the catalysts were reduced at 400 °C for 10 h by pure H_2_. The reaction was initiated by introducing mixed gases (H_2_: 71.3%; CO: 23.7%; Ar: 5.0%), with GHSV = 40 000 cm^3^ g^−1^ h^−1^. The effluent gas was on line analyzed using a gas chromatograph (GC-2014C, Shimadzu) equipped with a thermal conductivity detector (TCD) and flame ionization detector (FID).

## Results and discussion

### XRD

**Fig. 1:**
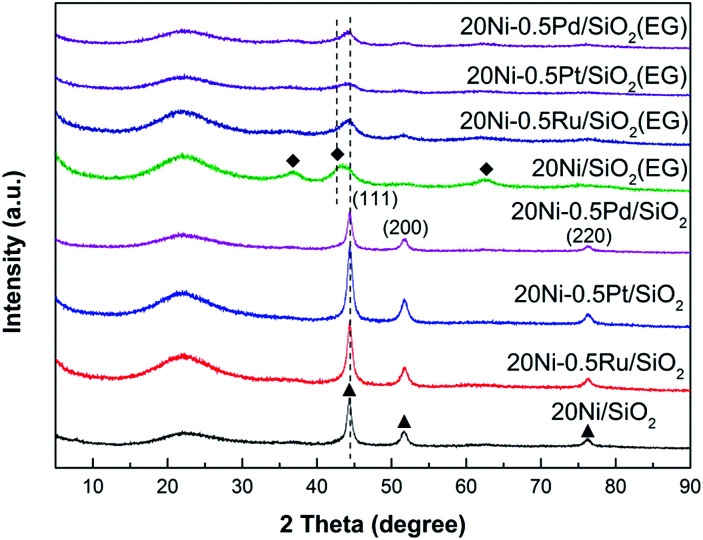
XRD patterns of various passivated Ni-based catalysts: (◆) NiO; (▲) Ni. The catalysts were reduced at 400 °C for 10 h using pure H_2_ and then passivated by 1% O_2_ in N_2_ at room temperature.

XRD was used to investigate the bulk crystalline structure of all Ni-based catalysts. The XRD patterns of various passivated samples are shown in Fig. 1. All samples exhibit a broad diffraction peak in the 2*θ* range between 10° and 30° which can be ascribed to the amorphous SiO_2_.^20^ The peaks at 44.5°, 51.7°, and 76.3° were the characteristic peaks of metallic Ni with a face-centered cubic structure (JCPDS# 87-0712). All the Ni-based catalyst prepared with non-pretreated silica support exhibits sharp and strong nickel peaks, and only metallic Ni is observed as the Ni species in all samples. In contrast, the nickel peaks of Ni-based catalyst prepared by ethylene glycol (EG) pretreated silica support were too broad and weak to calculate the crystalline size. This result suggests that the highly dispersed Ni catalysts were obtained due to the modified properties of the silica surface by EG pretreatment,^17^ resulting in lower crystalline size of supported metal, which would realize more active sites in the CO methanation reaction. Moreover, weak NiO diffraction peaks were detected in the 20Ni/SiO_2_ (EG) sample which exhibit the fcc-NiO phase (JCPDS no.78-0429) with typical reflections for the (111), (200), and (220) planes at 2*θ* = 37°, 43°, and 64°, respectively.^21^ This implies that the reduction of Ni oxide to Ni species in the EG-pretreated catalysts was difficult compared with the non-pretreated samples, due to the stronger metal–support interaction between the smaller crystalline size of NiO and silica support. However, this reducibility of the EG-pretreated catalysts can be enhanced by the addition of noble metal promoter. As shown in Fig. 1, for noble metal promoted samples, the peak assigned to the Ni oxide was disappeared, and the diffraction peaks assigned to metallic Ni appeared dominantly. It is generally known that, when a small amount of noble metal is added to a base metal oxide, dissociation of hydrogen takes place easily on the noble metal sites and the dissociated hydrogen can spill-over to the oxide support. Because such spill-over hydrogen is quite active, the reduction of the oxide can proceed easier than that for the pure oxide.^22,23^ Hence, in the present study, it is reasonable to consider that the reduction of NiO in noble metal promoted samples can be enhanced by the spill-over hydrogen from the noble metal to the Ni oxide. On the other hand, it is proposed that the NiO–support interaction will be weakened by the noble metal, which also enhanced the reduction of NiO to Ni.

**Fig. 2:**
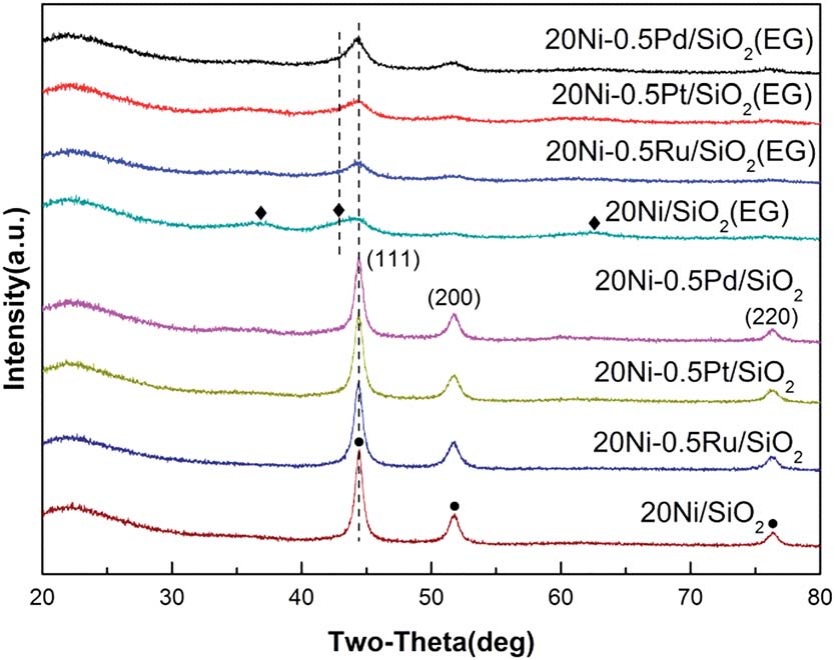
XRD patterns of various used Ni-based catalysts after 10 h CO methanation reaction. (◆) NiO; (●) Ni.

The XRD patterns of the Ni-based catalysts after CO methanation reaction are displayed in Fig. 2. It can be found that the intensity of the peak assigned to NiO in 20Ni/SiO_2_ (EG) was weakened obviously compared with that of the passivated sample, indicating that the reduction of NiO to Ni was continuously proceed during the reaction. The crystallite sizes of the Ni were calculated with the Scherrer equation using the parameters obtained from XRD, as compared in Table 1.

**Table 1:** The properties of various samples as prepared, after reduction and reaction

Catalysts	Ni particle size (nm)		Ni particle size (nm)		Dispersion^e^ (%)	CO_L_/CO_B_^f^	Surface M/Ni atomic ratio^g^
	XRD^a^	TEM^b^	XRD^c^	TEM^d^			
20Ni/SiO_2_	14.3	13.5	16.8	18.6	11.7	0.73	—
20Ni/SiO_2_ (EG)	N.A.	5.1	N.A.	5.4	28.3	1.32	—
20Ni–0.5Ru/SiO_2_	12.7	10.8	13.4	12.2	13.2	1.02	0.63
20Ni–0.5Ru/SiO_2_ (EG)	N.A.	4.5	N.A.	4.3	30.1	1.40	0.26
20Ni–0.5Pt/SiO_2_	12.8	11.5	13.0	12.9	13.0	1.05	0.24
20Ni–0.5Pt/SiO_2_ (EG)	N.A.	4.7	N.A.	5.5	29.5	1.37	0.18
20Ni–0.5Pd/SiO_2_	14.0	13.1	15.0	15.8	12.1	0.94	0.18
20Ni–0.5Pd/SiO_2_ (EG)	N.A.	5.9	N.A.	6.3	25.6	1.23	0.05

a Ni crystallite size as determined by X-ray diffraction of passivated samples.

b Ni crystallite size as determined by TEM of passivated samples.

c Ni crystallite size as determined by X-ray diffraction after CO methanation reaction.

d Ni crystallite size as determined by TEM after CO methanation reaction.

e Determined by hydrogen chemisorptions.

f The ratio of linearly adsorbed CO to bridge adsorbed CO, calculated by CO adsorption peak area in DRIFTS.

g Determined by XPS, the stoichiometric M/Ni atomic ratio of all catalysts is 0.025.

For the highly dispersed catalysts (20Ni/SiO_2_ (EG), 20Ni–0.5Ru/SiO_2_ (EG), 20Ni–0.5Pt/SiO_2_ (EG), and 20Ni–0.5Pd/SiO_2_ (EG)), the catalysts after reaction remain evident little change in Ni crystalline size compared with the passivated samples (Table 1), implying that the nanoparticles are stable to some extent upon reaction. Regarding 20Ni/SiO_2_ catalyst, the catalysts must have experienced sintering to some extent during reaction, because the metal crystal size increased from 14.3 nm before reaction (passivated sample) to 16.8 nm after reaction. It is worth noticing that the addition of noble metal did not prevent the occurrence of sintering during the reaction for all non-pretreated catalysts.

### TG/DTA

**Fig. 3:**
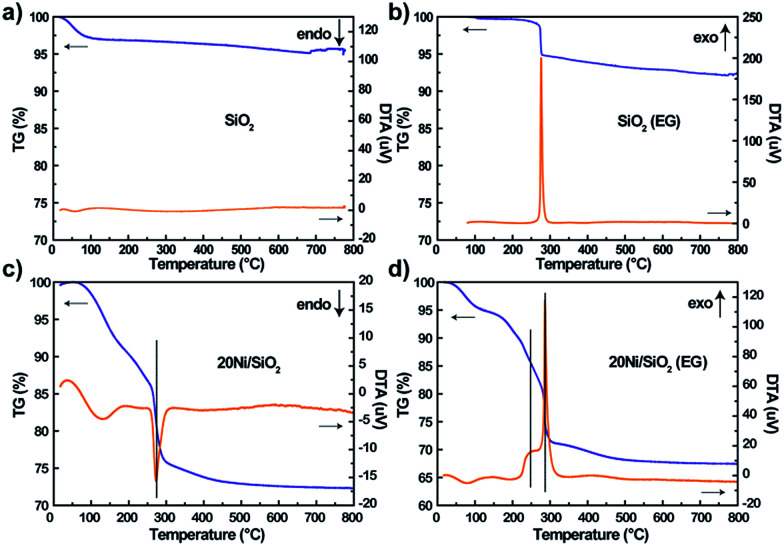
TG/DTA patterns of various precursors after drying at 120 °C for 12 h: (a) SiO_2_; (b) SiO_2_ (EG); (c) 20Ni/SiO_2_; (d) 20Ni/SiO_2_ (EG).

The precursors pretreated by EG or not were characterized by TG/DTA to examine the effect of adsorbed EG on the decomposition of impregnated nickel nitrate. As shown in Fig. 3a and b, the SiO_2_ (EG) sample exhibited one strong exothermic peaks at 276 °C due to the combustion of absorbed EG, while the non-pretreated silica support did not exhibit obvious weight loss. For the 20Ni/SiO_2_ and 20Ni/SiO_2_ (EG) precursors (Fig. 3c and d), the weight loss at low temperature (<200 °C) should be attributed to the evaporation of absorbed water. Meanwhile, it is clearly found that the 20Ni/SiO_2_ precursor exhibited a strong endothermic peak at 273 °C, which can be attributed to the decomposition of impregnated nickel nitrate. However, the DTA pattern of 20Ni/SiO_2_ (EG) exhibited two exothermic peaks at 259 °C and 286 °C, which should include a heat coupling process, namely, the decomposition of nickel nitrate and the combustion of the absorbed EG.

Hence, it is proposed that the heat uniformly released from thermal decomposing of EG during the calcination step will quickly absorbed by the precursor of catalyst to supply energy for decomposition of nitrates, which efficiently restrained the aggregation of the metal oxide, resulting in smaller particle size.

### TEM

**Fig. 4:**
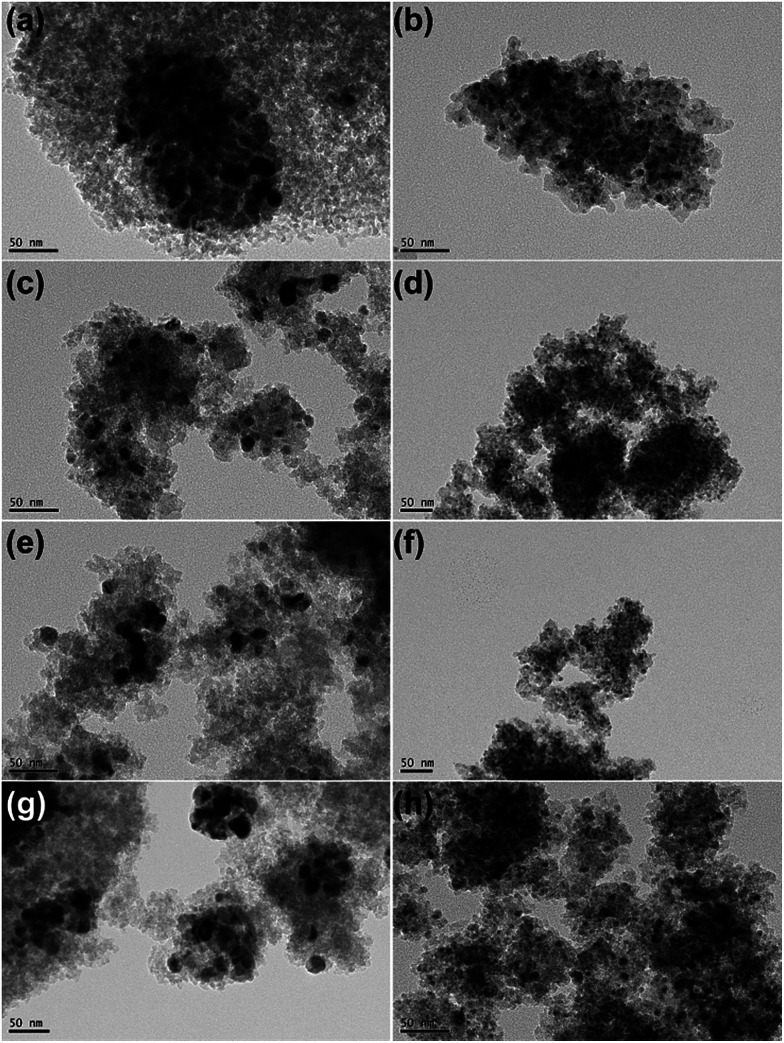
TEM images of all passivated Ni-based catalysts: (a) 20Ni/SiO_2_; (b) 20Ni/SiO_2_ (EG); (c) 20Ni–0.5Ru/SiO_2_; (d) 20Ni–0.5Ru/SiO_2_ (EG); (e) 20Ni–0.5Pt/SiO_2_; (f) 20Ni–0.5Pt/SiO_2_ (EG); (g) 20Ni–0.5Pd/SiO_2_; (h) 20Ni–0.5Pd/SiO_2_ (EG).

Fig. 4 shows TEM images of all passivated Ni-based catalysts. In the case of 20Ni/SiO_2_ catalyst, larger crystal particle aggregated into even larger clusters (Fig. 4a). The average nickel crystalline size are 13.5 nm, which are in good agreement with the XRD data, as tabulated in Table 1. In contrast, for all the catalysts pretreated by EG, significant differences in Ni particle size were observed (Fig. 4b, d, f and h). The 20Ni/SiO_2_ (EG) catalyst exhibited remarkable smaller particle size and distributed homogeneously (Fig. 4b); the average size of metal particle was *ca.* 5.1 nm, calculated from the statistical TEM data over 100 particles. The same trends were found for the 20Ni–0.5Ru/SiO_2_ (EG), 20Ni–0.5Pd/SiO_2_ (EG) and 20Ni–0.5Pt/SiO_2_ (EG) catalysts. Moreover, for the non-pretreated catalysts, it can be seen that the metal dispersion over the noble metal promoted catalysts is slightly higher than that over unpromoted catalysts, as shown in Fig. 4 and Table 1. It is proposed that there is a kind of interaction between Ni and noble metal, and this synergetic effect may enhance the metal dispersion.^24^ Therefore, the significantly increased Ni dispersion of EG pretreated catalysts with noble metal promoter should be induced by EG pretreatment of silica support instead of added noble metals.

**Fig. 5:**
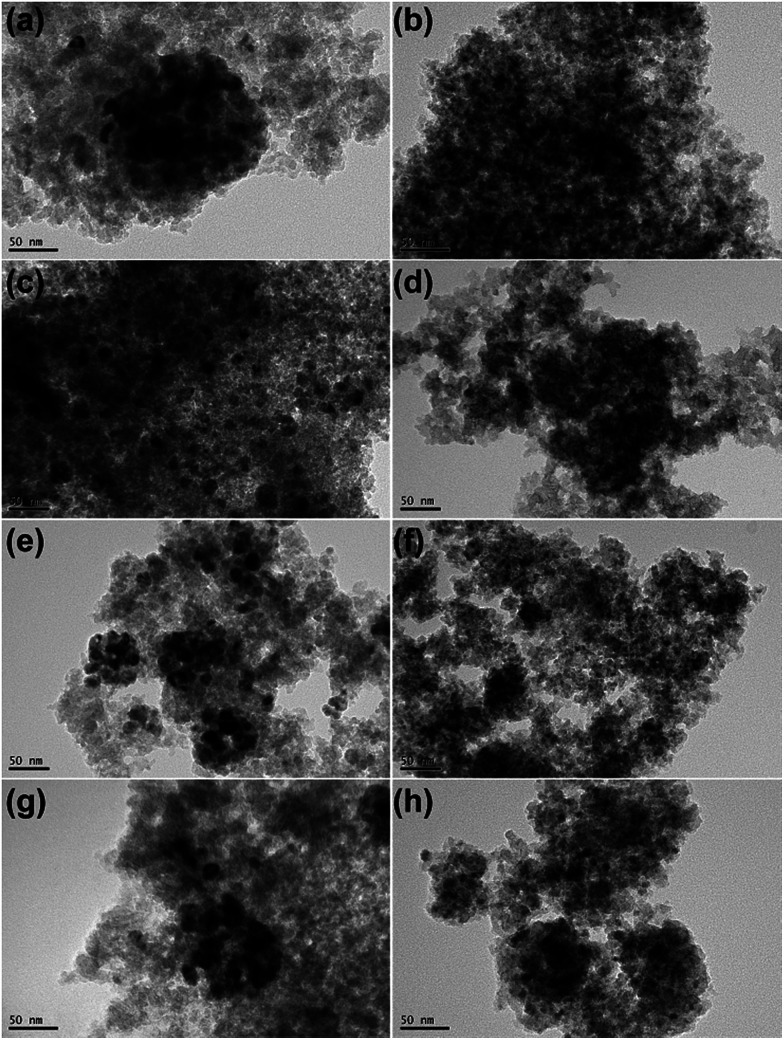
TEM images of all used Ni-based catalysts after 10 h reaction: (a) 20Ni/SiO_2_; (b) 20Ni/SiO_2_ (EG); (c) 20Ni–0.5Ru/SiO_2_; (d) 20Ni–0.5Ru/SiO_2_ (EG); (e) 20Ni–0.5Pt/SiO_2_; (f) 20Ni–0.5Pt/SiO_2_ (EG); (g) 20Ni–0.5Pd/SiO_2_; (h) 20Ni–0.5Pd/SiO_2_ (EG).

**Fig. 6:**
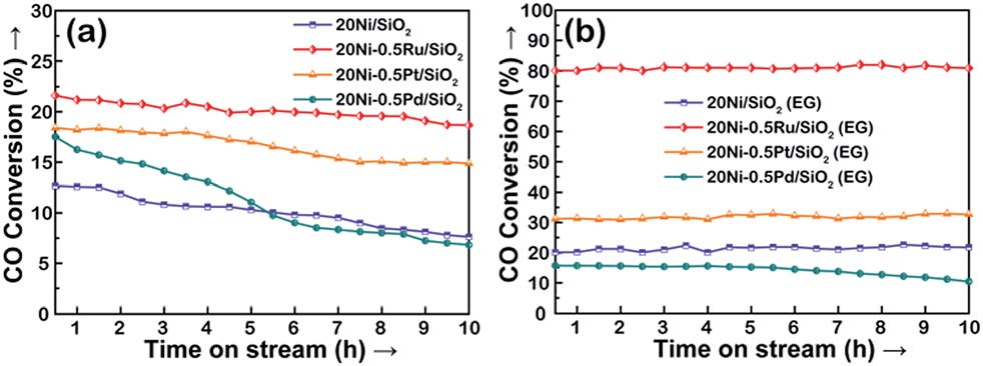
CO conversion as a function of time on stream for the Ni-based catalysts: (a) non-pretreated; (b) EG-pretreated. Reaction conditions: 275 °C, 1 bar, H_2_/CO = 3, GHSV = 40 000 cm^3^ g^−1^ h^−1^, weight of catalyst = 0.1 g.

The TEM images of various used Ni-based catalysts after CO methanation reaction were compared in Fig. 5. In the case of 20Ni/SiO_2_ catalyst, the average NiO_*x*_ size increased from 13.5 nm for passivated sample to 18.6 nm, while for 20Ni/SiO_2_ (EG) the average size after reaction was 5.4 nm, which is similar with that of passivated sample (5.1 nm). This is in good agreement with those obtained from XRD. From these results, it is concluded that the catalyst with a large initial particle size suffered a higher extent of sintering compared to a catalyst with a smaller size, and thus leads to rapid deactivation with time on stream (TOS) during the reaction. In contrast, all EG-pretreated catalysts showed a stable catalytic activity for 10 hours, as shown in Fig. 6. It is well known that the nanoparticles in the highly dispersed catalysts are stable to some extent upon reaction.^19,25^ Hence, the low deactivation rate of these highly dispersed catalysts indicate that the smaller Ni clusters are less prone to deactivation, since the smaller Ni particles can present more available sites for the catalytic reaction and reduce the carbon deposition.^26–28^ On the other hand, the stronger metal–support interaction for the smaller Ni particles can also slow down Ni nanoparticle sintering during the highly exothermic methanation process.^29,30^

### XPS

**Fig. 7:**
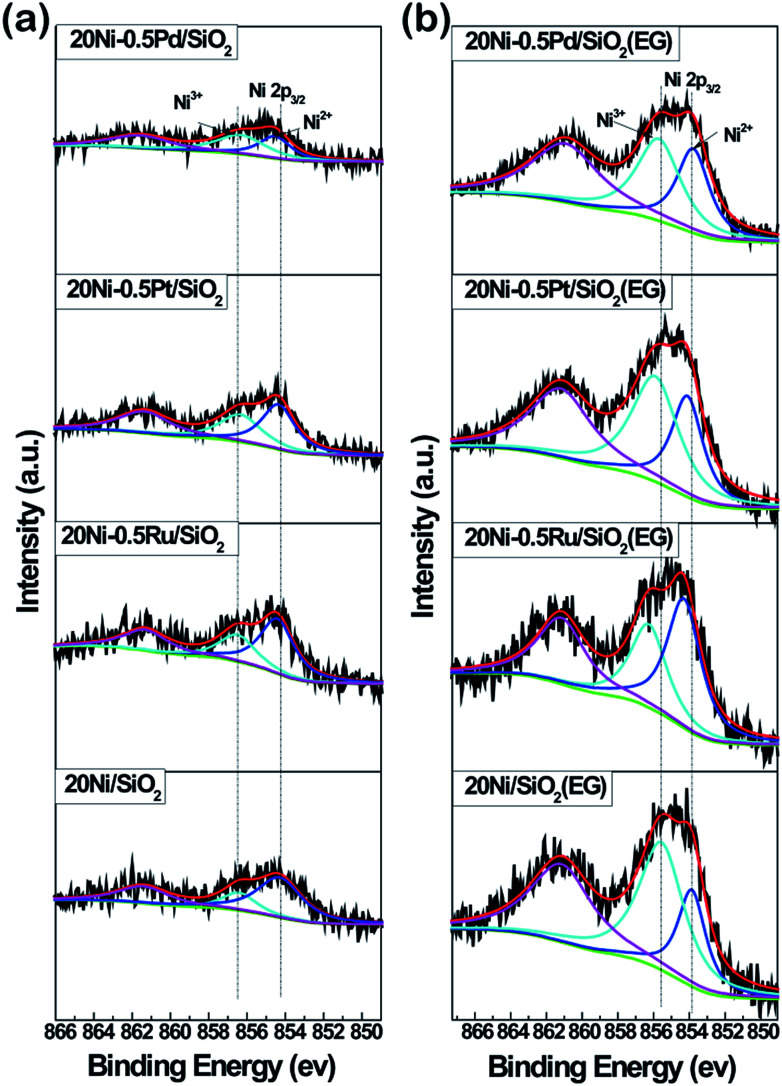
XPS spectra of Ni 2p core level recorded from Ni-based catalysts as prepared.

XPS analysis of all Ni-based catalysts as prepared was carried out to examine the chemical state of surface Ni species in the samples, as shown in Fig. 7. Two fitted peaks at binding energies of 854.7 eV and 856.6 eV were observed in the high resolution spectrum of Ni 2p_3/2_, along with the broad satellite centered at around 860 eV,^31,32^ which is characteristic of NiO species.^20,33^ In addition, it can be seen that the intensity of Ni 2p_3/2_ in all EG-pretreated samples is stronger than that in non-pretreated samples, suggesting the presence of a higher Ni density on the surface of EG-pretreated Ni-based catalysts due to the smaller Ni particles. These results are in agreement with the XRD and TEM analysis. Meanwhile, it was found that the binding energy of the Ni 2p_3/2_ peak in 20Ni/SiO_2_ (EG) sample was lower than that for 20Ni/SiO_2_, indicating that a strong interaction between Ni and support was formed due to the smaller Ni particles.

However, in the case of noble metal promoted catalysts, the electron transfer between Ni and support was disturbed by promoters. As shown in Fig. 7a, the binding energy of Ni 2p_3/2_ in Ni samples with promoter is slightly higher (0.2 eV) than that of 20Ni/SiO_2_. In the case of EG modified samples (Fig. 7b), the binding energy was further shift to higher energy. This implies that there is an electron transfer between Ni and noble metal atoms. In bimetallic catalysts, the electron transfer between metals has been proposed previously, such as Ti–Pt^34^ and Ru–Mo,^35^ where electrons transfer from Ti to Pt or from Mo to Ru. It seems that the electron usually transfers from low electronegative elements to high electronegative elements.^36^ In this work, the higher Ni 2p binding energies in noble metal promoted Ni catalysts relative to non-promoted Ni catalysts indicate that electron transfers from the low electronegative Ni element to the high electronegative noble metal element occur. Moreover, the transferred charge amount is largest for 20Ni–0.5Ru/SiO_2_ (EG) catalyst and decreases in the order of 20Ni–0.5Ru/SiO_2_ (EG) > 20Ni–0.5Pt/SiO_2_ (EG) > 20Ni–0.5Pd/SiO_2_ (EG) catalyst. This may imply that there are strong interactions between noble metal and Ni atoms, which could enrich surface noble metal and enhance the activation of adsorbed CO. This was confirmed by the surface atomic ratio of noble metal to Ni, as shown in Table 1.

### H_2_-TPR

**Fig. 8:**
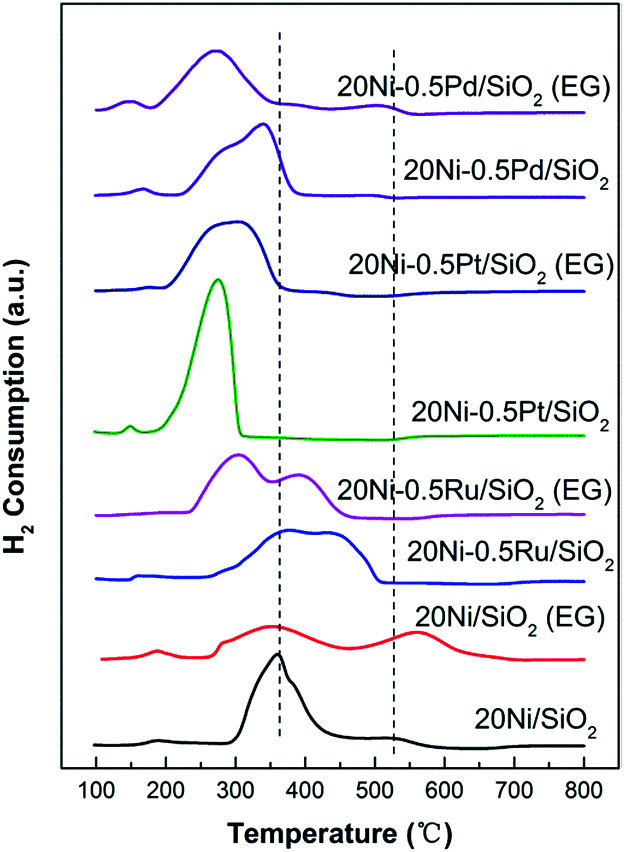
H_2_-TPR spectra of various Ni-based catalysts.

The reduction behavior of the silica supported Ni-based catalysts was measured by temperature programmed reduction (TPR). As shown in Fig. 8, the 20Ni/SiO_2_ catalyst shows two distinct H_2_ consumption peaks with different areas between 200 and 700 °C. The first peak with larger area can be attributed to the reduction of NiO particles that weakly interact with SiO_2_ support, and the peak around 520 °C suggests the existence of NiO particles that strongly interact with the support on this 20Ni/SiO_2_ sample.^37,38^ The TPR profile of 20Ni/SiO_2_ (EG) differs significantly from that of 20Ni/SiO_2_. The first reduction region gradually shifted to lower temperature while the second peak shifted to a higher temperature with the increased intensity. It is generally accepted that smaller NiO particles can be more easily reduced than larger ones, due to the higher probability of contact with H_2_ during the reduction.^39,40^ Hence, the NiO species in 20Ni/SiO_2_ (EG) catalyst is more easily reduced at low temperature than that on 20Ni/SiO_2_ catalyst, due to the smaller NiO crystalline size, as supported by XRD and TEM analysis. However, a strong interaction between NiO and a support can cause a shift of the TPR peak to a higher temperature. As confirmed by XPS result (Fig. 7), smaller metal oxide nanoparticles and a support can strongly interact, which makes it difficult to interpret the TPR data considering particle size of the metal oxide alone. The shifting of the second peak to a higher temperature for 20Ni/SiO_2_ (EG) compared with that of 20Ni/SiO_2_ can be ascribed to the difficulty in reducing NiO, which strongly interact with the silica support.

Furthermore, in the case of noble metal promoted catalysts, all TPR profiles evidently shifted to lower temperature ranges with multiple peaks compared with those for unpromoted Ni catalysts shown in Fig. 8. The decrease of the reduction temperature of nickel oxides for 20Ni–0.5Ru/SiO_2_, 20Ni–0.5Pt/SiO_2_, and 20Ni–0.5Pd/SiO_2_ sample is caused by the existence of noble metal, which has a role of the porthole of the hydrogen spill-over as reported in the other literatures.^22,23^ In addition, in the case of noble metal promoted catalyst modified by EG, the peak temperatures of TPR profiles further shifted to lower temperature to 303, 301, and 271 °C correspondingly to 20Ni–0.5Ru/SiO_2_ (EG), 20Ni–0.5Pt/SiO_2_ (EG) and 20Ni–0.5Pd/SiO_2_ (EG), respectively, due to the smaller crystalline size and moderate Ni–support interaction. This result indicates that the hydrogen reduction was enhanced by the promotion of smaller Ni particles and hydrogen spill-over effect. The high reducibility for the EG-modified noble metal promoted catalyst may contribute to the higher CO methanation activity.

### *In situ* DRIFTS

**Fig. 9:**
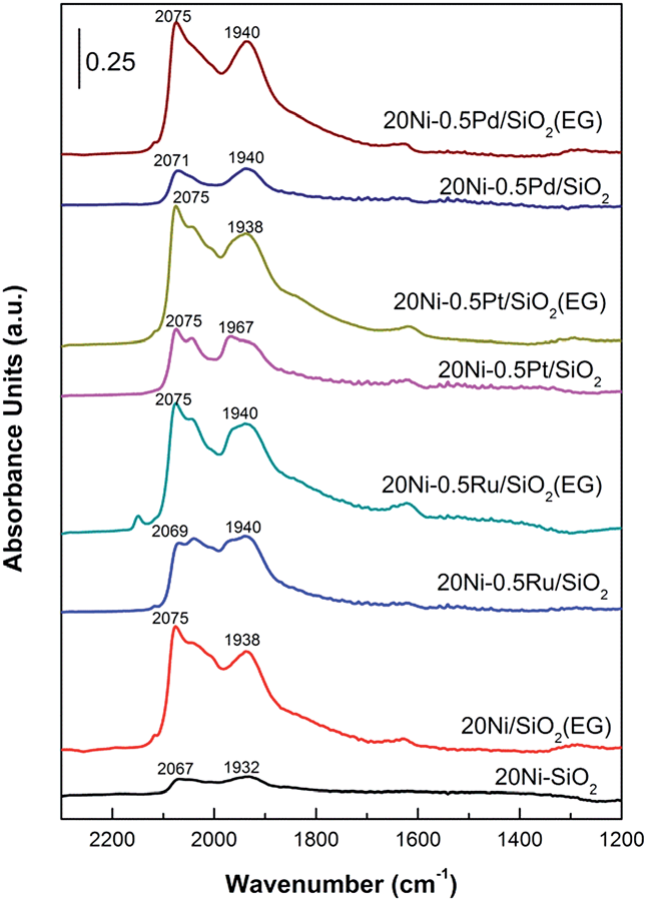
*In situ* CO-DRIFT spectra of various Ni-based catalysts.

The Ni properties of all catalysts were probed using *in situ* DRIFTS in which 15 mg of passivated catalysts were packed in the reactor chamber and supported by a wire mesh. The passivated catalysts were first reduced *in situ* at 400 °C for 1 h under 30 mL min^−1^ of H_2_ and purged with N_2_ to remove the physisorbed hydrogen before 95% CO/5% N_2_ (30 mL min^−1^) were introduced in to the reaction chamber to investigate the Ni properties of catalyst. *In situ* IR spectra were obtained at room temperature. As shown in Fig. 9, the signal probably entails linear CO species (CO_L_) on metallic Ni as well that are reported to exhibit signals at 2075–2050 cm^−1^.^21,41^ The band of CO adsorbed on Ni metal in linear mode shifted from 2032 cm^−1^ for 20Ni/SiO_2_ catalyst to 2040 cm^−1^ for 20Ni/SiO_2_ (EG) catalyst here.^42^ The blue shift of the Ni–CO band is due to the increase in the dipole–dipole interaction which occurs because the Ni surface CO coverage increases on the smaller Ni,^21,43^ according to the data of XRD and TEM (Table 1), indicated that the EG modified support promoted the dispersion of supported nickel. Meanwhile, strong CO adsorption strength of the EG modified catalysts implied that the formation of smaller Ni particles enhance CO dissociate and in turn promote the formation of carbonyl species, which produces methane as desired product. For noble metal promoted catalysts, the peak of the linear adsorbed CO was also significantly stronger than that of unpromoted 20Ni/SiO_2_ catalyst, and EG pretreatment further increase the intensity.

The peak at 1940 cm^−1^ is the result of bridge-type CO species (CO_B_) coordinated to nickel sites (Ni(CO)_2_) on the surface of the catalysts.^21,44^ For 20Ni/SiO_2_ (EG) catalyst, the peak of bridged adsorbed CO was stronger than that of 20Ni/SiO_2_ catalyst, as the higher dispersion and reducibility of supported nickel formed more reactive nickel sites. More importantly, the ratio of CO_L_/CO_B_ was calculated from peak area of adsorbed CO, as shown in Table 1. It was reported that CO linearly coordinated to Ni is less strongly bonded than bridge bonded CO and is also more reactive toward CO methanation.^21^ On the contrary, CO bridge-type adsorbed on crystalline sites seems to be less reactive as it is rather accumulated than dissociated.

Consequently, the higher ratio of CO_L_/CO_B_ means to the higher reaction activity in CO methanation reaction. It can be found that the ratio of CO_L_/CO_B_ increased significantly after EG pretreatment for all samples, and the addition of noble metal promoter also improved this. Hence, it is proposed that the catalytic performance will be significantly improved due to the synergetic effect of noble metal promoter and small Ni particle.

### CO methanation performance

The catalytic performances of Ni-based catalysts prepared by wet impregnation method in CO methanation were studied at temperatures ranging from 200 to 280 °C at a GHSV of 40 000 cm^3^ g^−1^ h^−1^, a H_2_/CO molar ratio of 3 : 1 and atmospheric pressure.

For all reaction temperatures, CH_4_ is the major product and very small amount of CO_2_ and light hydrocarbons (C_2_–C_3_) was measured as an additional product.

**Fig. 10:**
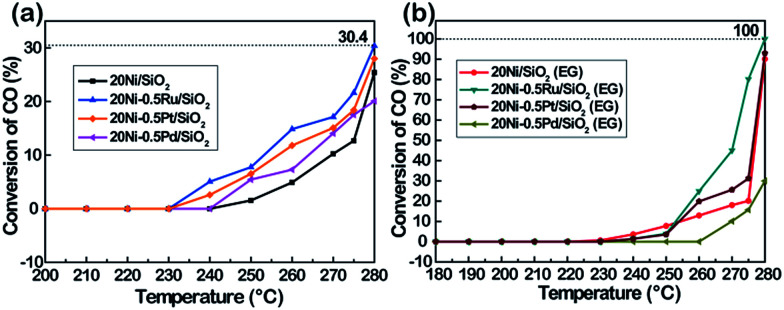
The CO conversion of various Ni-based catalysts. Reaction conditions: *P* = atmospheric pressure, CO/H_2_ = 1 : 3, GHSV = 40 000 cm^3^ g^−1^ h^−1^.

As presented in Fig. 10, it can be seen that the conversion of CO over the catalyst increases with increasing reaction temperature. Among the tested catalysts, 20Ni/SiO_2_ exhibits the lowest activity and the conversion of CO is lower than 13% even at 275 °C. In contrast, the 20Ni/SiO_2_ (EG) catalyst realized the maximum of CO conversion of 20.2% at 275 °C. The higher catalytic activity of 20Ni/SiO_2_ (EG) can be ascribed to the smaller Ni particle size and higher reducibility, which would increase the amount of surface active sites.

As shown in Fig. 10a, in the case of non-pretreated samples, the unpromoted 20Ni/SiO_2_ catalyst shows lower CO conversion than noble metal promoted Ni catalysts, while incorporating Ru, Pt, or Pd into the Ni/SiO_2_ catalyst significantly increased methanation activity and followed the order of Ru > Pt > Pd. This promotion effect was more significant for the EG-pretreated samples except 20Ni–0.5Pd/SiO_2_ (EG). It is observed from Fig. 10b that the CO conversion of 20Ni–0.5Ru/SiO_2_ (EG) reached as high as 80.2% at 275 °C, and the catalytic activity of 20Ni–0.5Pt/SiO_2_ (EG) also increased to 31.2%. It is proposed that the dispersion of Ni on the support was increased for the EG-modified noble metal promoted Ni catalysts, then the effect of hydrogen spill-over by the existence of noble metal could progress more efficiently.^37^ Moreover, a number of studies have shown that Ru catalysts are very active for methanation and can have high activities even at low temperatures.^5,45,46^ For instance, Vannice^47^ reported that the specific activity of Ru/Al_2_O_3_ for CO hydrogenation is about one order of magnitude higher, compared to that of Al_2_O_3_-supported Rh or Pd. Therefore, adding small amount of Ru in Ni catalyst is a promising way to promote methanation reaction at lower temperature.^48^ However, the lower activity of Pd-promoted catalyst can be ascribed to the SMSI (strong metal–support interaction) behavior exist in highly dispersed 20Ni–0.5Pd/SiO_2_ (EG) catalyst, and one consequence of this SMSI effect is a severe inhibition of CO and H_2_ chemisorption on well-dispersed Pd particles.^49^

**Fig. 11:**
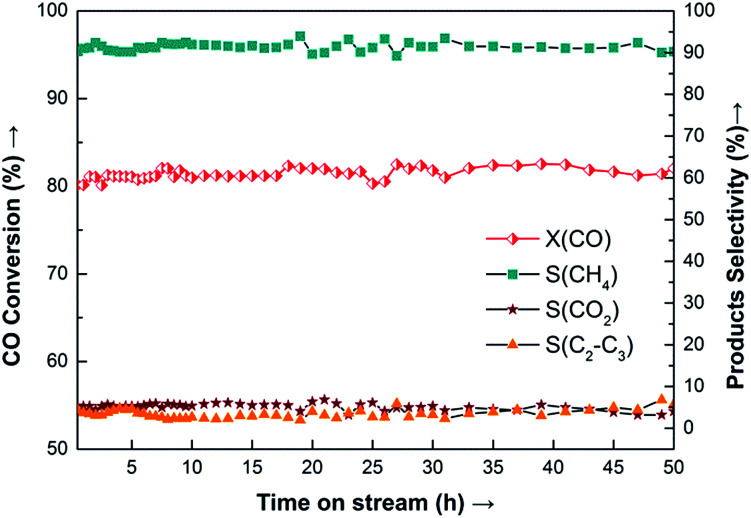
Long-term stability test of 20Ni–0.5Ru/SiO_2_ (EG) catalyst for CO methanation. Reaction conditions: *T* = 275 °C, *P* = atmospheric pressure, CO/H_2_ = 1 : 3, GHSV = 40 000 cm^3^ g^−1^ h^−1^.

Because the 20Ni–0.5Ru/SiO_2_ (EG) catalyst showed the highest methanation activity, the stability was investigated. Results obtained are presented in Fig. 11, where the conversion of CO (X_CO_) and the selectivity of methane (S_CH4_) and other products are plotted as the function of time-on-stream. It is observed that 20Ni–0.5Ru/SiO_2_ (EG) exhibits excellent stability for more than 50 h-on-stream, with conversion of CO being about 81% and selectivity to methane around 91%, because the synergetic effect of small Ni particle and noble metal promoter prevent the sintering of supported Ni and carbon deposition.

## Conclusions

The highly dispersed SiO_2_ supported Ni-based catalysts were prepared by EG-modified wet impregnation method. This catalyst was far superior for CO methanation than the conventional Ni-based catalysts due to the high Ni dispersion and metal–support interaction. In addition, the effects of noble metal promoter (Ru, Pt, Pd) on the catalytic performance of SiO_2_-supported nickel catalysts were investigated. It was found that the catalytic activity was improved dramatically by adding noble metal, due to the enhanced reducibility by hydrogen spill-over, moderate Ni–support interaction, and intrinsic hydrogenation capacity of noble metal. For all experimental conditions investigated, Ru are significantly more active than Pt and Pd. Overall, the highly dispersed 20Ni–0.5Ru/SiO_2_ (EG) catalyst that is simply pretreated by ethylene glycol shows a high resistance for catalyst deactivation with high catalytic performance due to the synergistic effects of small Ni particles and noble metal promoter, and this catalyst can be one of the best catalysts for applying CO methanation reaction.

## Conflicts of interest

There are no conflicts to declare.

## Supplementary Material
